# Comparison of Different Hemostatic Net Applications in a Rat Dorsal Skin Flap Model

**DOI:** 10.1093/asj/sjaf010

**Published:** 2025-01-21

**Authors:** Bilge Kaan İsmail, Kemal Fındıkçıoğlu, Serhat Şibar, Çiğdem Elmas

## Abstract

**Background:**

Although several articles have discussed the use of hemostatic nets to close dead spaces, no in vivo experimental studies have examined simultaneously the histology and tissue perfusion of these techniques.

**Objectives:**

The aim of this study was to compare variations of the hemostatic net technique commonly used in current practice.

**Methods:**

Two different hemostatic net suturing techniques and 2 suture removal times were tested, with a control group for comparison. In a modified McFarlane flap model, hemostatic net sutures were placed in either a vertical or horizontal pattern. Suture removal times were set at 60 hours and 7 days. Perfusion in the proximal, middle, and distal parts of the flap was assessed by SPY-assisted immunofluorescence angiography (Novadaq, Kalamazoo, MI) at 0 minutes, 60 hours, and 7 days after the first surgery. The rat dorsal flap was photographed in a standardized manner 1 week postsurgery. Flap survival areas were calculated as a percentage using ImageJ software (US National Institute of Health, Bethesda, MD). On day 20, all rats were killed and sent for histological examination.

**Results:**

There was no statistically significant difference in macroscopic flap survival between the groups (*P* > .05). Group 5 (control) was statistically different with lower neovascularization scores than the other groups in all 3 segments (*P* < .01).

**Conclusions:**

Hemostatic nets may improve neovascularization at the flap base but do not significantly affect overall flap survival.

In many plastic surgery procedures, tissue layers are separated through surgical dissection. After the procedure, these layers gradually adhere back together, helping to close the empty space created (known as the “dead space”). However, if left unaddressed, blood or lymphatic fluid can collect in this space, forming a hematoma or seroma. To prevent these complications and promote faster healing, various methods are commonly used, including drainage, pressure dressings, corsets, and both internal and external sutures, which help secure the tissues in place.

Several articles have discussed the use of a hemostatic net to close dead spaces, but no in vivo experimental studies have examined simultaneously the histology and tissue perfusion of these techniques. Hemostatic nets are widely used by surgeons in facelifts, brow lifts, neck lifts, and similar procedures that require management of dead space. It is thought to effectively prevent hematoma formation and enhance tissue fixation.^1,2^ To avoid permanent skin scarring, the sutures applied with this technique are typically removed, usually on the second or third day postsurgery. However, most data supporting the use of hemostatic nets are based on personal experience, and only a few published studies exist.^1,3,4^

Our aim was to compare the variations of the hemostatic net technique, specifically the external continuous skin sutures commonly used in recent practices, by applying them in a modified McFarlane caudally based rat dorsal skin flap model.^1^ These variations include differences in suture orientation (vertical vs horizontal) and suture removal timing (60 hours vs 7 days), with a control group included for comparison.

The modified McFarlane flap model has been previously validated by histological, imaging, and observational methods, and is a widely used experimental model for studying flap viability and vascular dynamics.^5,6^ It involves creating a 9 × 3 cm^2^ caudally based skin flap on the dorsal region of a rat, with the flap fully elevated and then returned to its original position. Sutures for the hemostatic net are placed either vertically or horizontally according to the flap's vascular axis. The sutures are removed after 60 hours or 7 days. The 60-hour point represents the average suture removal time in current clinical practice; the 7-day point represents the typical removal time for areas such as the scalp, where scarring is less of a concern.^1,3,4,7,8^ This study thus allows for observation of how different suture patterns and removal timings affect flap vascularity and histology in both vertical and horizontal placements. To assess collagen production during wound healing, a follow-up period of 20 days was chosen.

## METHODS

This study, conducted between August 2023 and June 2024, received ethical approval from the Gazi University Local Ethics Committee for Animal Experiments (G.Ü.ET-23.019). Thirty male Wistar Albino rats, each at least 12 weeks old and of similar weight, were used. Under general anesthesia, the rats were positioned supine. To standardize suture placement, the prominence of the rat hip was identified as the proximal boundary of the flap along the midline, and a 9 × 3 cm^2^ area was marked for incision. After sterilizing the surgical field, a 9 × 3 cm^2^ caudally based modified McFarlane dorsal skin flap was carefully elevated.

To perform the hemostatic net technique, the skin flap was sutured with 5-0 sharp polypropylene sutures (19 mm; Ethicon Inc., Raritan, NJ), starting either from the proximal to the distal end (vertical direction) or from the left lateral to the right lateral side (horizontal direction). The incision line was then primarily sutured at intervals of 1 cm.

### Experimental Groups

**Figure 1.**
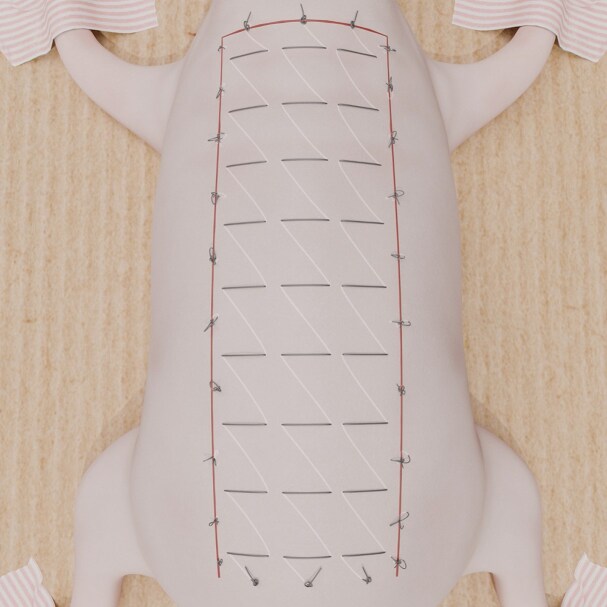
Vertically oriented hemostatic net technique.

In Group 1, net sutures were applied from the caudal to the cranial (vertical direction) (Figure 1). In each suture, tissues were covered approximately 0.8 cm horizontally and 1 cm vertically from the base to the distal end of the flap, and the suture was knotted upon reaching the distal edge. This creates a suture column along the midline, with 2 additional suture columns placed parallel to it. The flap was secured to its bed at the incision line, and the net sutures were removed after 60 hours.

**Figure 2.**
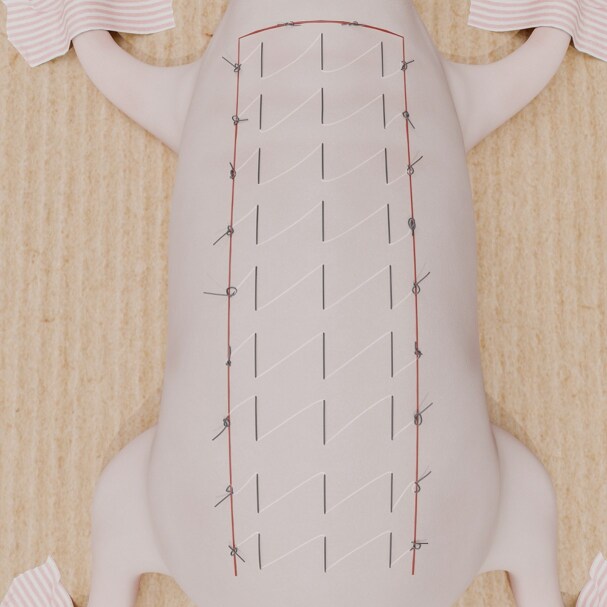
Horizontally oriented hemostatic net technique.

In Group 2, net sutures were applied from left lateral to right lateral (horizontal direction) (Figure 2). In each suture, the tissues were covered 0.8 cm vertically and 1 cm horizontally, starting from the left lateral base of the flap and moving to the right lateral edge, where the suture was knotted. This forms a proximal suture line, with 8 additional lines placed parallel to it. The flap was secured to its bed at the incision line, with net sutures removed after 60 hours.

In Group 3, the same procedure as in Group 1 was followed, but net sutures were removed on Day 7. In Group 4, the same procedure as in Group 2 was applied, but net sutures were removed on the Day 7. In the control group (Group 5), the flap was sutured primarily along the incision line. In all groups skin sutures were removed on Day 7 (Table 1).

**Table 1.** All Groups in the Study

Groups	Time for hemostatic net suture removal	Hemostatic net direction of progression	No. of animals
Group 1	60 hours	Vertical	6
Group 2	60 hours	Horizontal	6
Group 3	7 days	Vertical	6
Group 4	7 days	Horizontal	6
Group 5 (control)	No hemostatic net suture	No hemostatic net suture	6

Perfusion was evaluated using SPY-assisted immunofluorescence angiography (Novadaq, Kalamazoo, MI) at 0 hours, 60 hours, and 7 days postsurgery. For this procedure, each rat's tail vein was catheterized, and a diluted indocyanine green solution (ICG; 25 mg/100 mL saline) was administered at an appropriate dose (0.3 mL) to obtain perfusion data. On Day 20 of the experiment, all rats were killed, and skin samples, including the base tissue, were sent for histological examination. For histology, 6-cm sections were prepared starting at the flap base and extending through the midline, with evaluations conducted at proximal, middle, and distal points at 2-cm intervals.

### Flap Perfusion Measurement With the SPY Fluorescence Angiography Device

**Figure 3.**
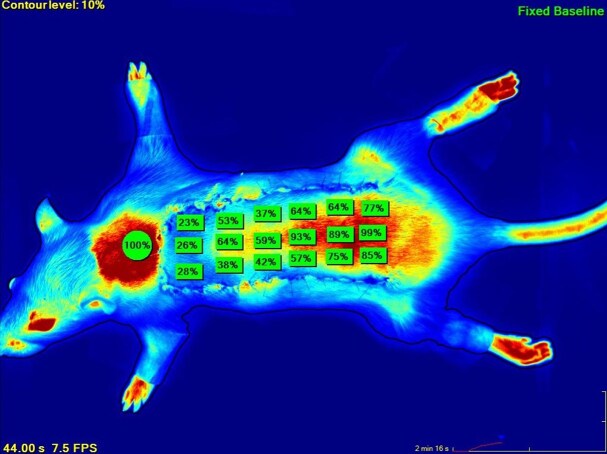
SPY-Q perfusion scores.

Perfusion recordings of the dorsal flaps were conducted for 3 minutes, after which the captured image was used for analysis. The rat dorsal flap was divided into 3 zones: proximal, middle, and distal. Perfusion values were calculated as percentages by SPY-Q software, with the nuchal region chosen as the reference point and defined as 100% perfusion (Figure 3). Each perfusion zone was divided into 6 equal areas of 1 × 1.5 cm^2^, and measurements were taken from the center of each area. The average of these values provided a mean perfusion score for each zone.

### Macroscopic Examination in Digital Media

One week after the surgical procedure, the modified McFarlane flaps from the dorsal area of each rat were photographed in a standardized manner. After digitization, flap survival areas were calculated as percentages by ImageJ software (US National Institutes of Health, Bethesda, MD).

### Hematoxylin-Eosin Staining

The stained sections were examined under a light microscope (Leica DM 4000, Wetzlar, Germany), and the images were digitized. Hematoxylin-eosin–stained sections from the proximal, middle, and distal parts of the 6 cm flap were evaluated. For each part, 3 areas with the highest neovascularization (hot spots) were selected under ×10 and ×20 objectives, totaling 9 areas per graft. New vascular lumens were counted in these areas under an ×40 objective. The average number of vessels in the 3 selected areas for each flap section was calculated and recorded as the mean number of newly formed vessels for that section.

### Masson's Trichrome Painting

The number and density of newly formed collagen fibers were assessed by Masson’s trichrome staining. Staining intensity was scored as follows: 1+ (faint), 2+ (moderate), and 3+ (strong). Strong dark-blue staining, indicating mature collagen tissue, was scored as 3+, pale grayish-blue as 1+, and intermediate staining as 2+.

### Statistical Analysis

Statistical evaluation was performed with IBM SPSS 20.0 software (SPSS Inc., Chicago, IL). The test for conformity to normal distribution was evaluated by the Shapiro-Wilk test. Numerical variables showing normal distribution were expressed as mean [standard deviation], numerical variables not showing normal distribution were expressed as median (25th-75th quartiles), and categorical variables were expressed as frequency (percentages). When the number of independent groups was more than 2, one-way analysis of variance (ANOVA) was used for numerical variables with normal distribution and Tukey test was used for post hoc analysis. Repeated-measures ANOVA was used to compare more than 2 dependent groups. *P* < .05 was considered sufficient for statistical significance.

## RESULTS

### Macroscopic Findings

No animals were lost during the experiment, and no complications occurred. Additionally, no macroscopic differences were observed among the rats throughout or at the end of the experiment.

### Histological Findings

To assess neovascularization, a 6-cm section from the proximal side of the flap was divided into three 2-cm segments (proximal, middle, distal), excluding the necrotic distal area. All groups showed a decrease in vessel count from proximal to distal. Post hoc analysis indicated that Group 5 (control) was statistically different with lower scores than the other groups in all 3 segments (*P* < .01). In each group, the highest vessel count was in the proximal segment and the lowest in the distal.

Masson’s trichrome staining for collagen revealed no statistically significant differences between groups (*P* > .05). Additionally, no significant histological differences were found among the groups in terms of edema, inflammation, hemorrhage, or connective tissue fibroblast density (*P* > .05).

### SPY Fluorescence Imaging Results

Statistical comparisons of perfusion based on SPY imaging were conducted for the proximal, middle, and distal parts of the flap at 0 minutes, 60 hours, and 7 days postsurgery. A decrease in perfusion from proximal to distal was observed in all groups.

For the proximal part, a significant difference was found between the 0-minute and 7-day measurements in Group 1 (*P* < .01) and in Group 5 (*P* < .05). No significant differences were noted in other pairwise comparisons (*P* > .05).

For the middle part, Groups 1, 2, 3, and 5 showed significant differences between the 0-minute and 7-day measurements, as well as between the 60-hour and 7-day measurements. In Group 4, only the comparison between the 0-minute and 7-day measurements was significant (*P* < .05).

For the distal part, all groups showed a statistically significant difference between the 60-hour and 7-day measurements (*P* < .01).

### Macroscopic Findings

There was no statistically significant difference in macroscopic flap survival rates between the groups (*P* > .05). Additionally, no significant correlation was found between macroscopic flap survival rate and overall new vessel formation (*P* > .05).

## DISCUSSION

The effects of the hemostatic net on skin perfusion have mostly been evaluated in clinical and cadaveric studies.^1,3,9,10^ This study is the first to examine the hemostatic net experimentally in vivo using histological methods and SPY fluorescence imaging. In random skin flaps, such as those used in facelift surgeries, indirectly connected vessels in the subdermal vascular plexus are critical for vascularization because they are the only preserved vessels during subcutaneous dissection.^11^

A common concern with hemostatic net use is that suture tension may negatively impact skin flap circulation due to ischemic effects. However, in a clinical study of 405 facelift patients, no adverse effect on ischemia or necrosis was reported.^1^

Xiong et al observed a horizontal subdermal vascular plexus in the malar region and a vertical plexus in the forehead.^12^ In this study, vertically oriented hemostatic nets were used in Groups 1 and 3, aligning with the vascular extension, and horizontal nets were used in Groups 2 and 4, perpendicular to it. Results showed no significant difference in flap survival between groups, indicating that the orientation of the hemostatic net does not impact vascular circulation.

ICG is increasingly used in facelift surgeries because intraoperative ICG fluorescence imaging can help to assess the risk of skin necrosis through perfusion scores.^13^ In a study of 8 patients undergoing Mustarde flap repair post–skin malignancy, SPY imaging before and after hemostatic net application showed no significant effect of the net on vascularity, with a trend toward reduced ischemia and necrosis as noted by Auersvald.^1,9^ A similar effect was observed with progressive tension sutures in facelift surgery, likely due to a more even tension distribution across tissues.^14^

In this study, SPY imaging scores for the proximal, middle, and distal parts of the flap were recorded at 0 minutes, 60 hours, and 7 days postsurgery. No significant differences were observed in SPY perfusion scores across regions or groups over time. SPY values consistently decreased from proximal to distal in all groups. For the proximal part, SPY scores increased from Minute 0 to Day 7 in all groups, with a significant rise in Group 1 and the control group. For the middle part, SPY scores rose from Minute 0 to Day 7 and from Hour 60 to Day 7 in all groups except Group 4, where a significant increase was only seen from Minute 0 to Day 7. The absence of a SPY score increase in Group 4 may suggest a disadvantage with the combination of horizontal application and Day-7 removal. The horizontal pattern might be considered a disadvantage because it is perpendicular to the primary vascular axes in the dorsal skin flap model. This orientation may create localized compression on the subdermal vascular plexus, potentially restricting blood flow and contributing to reduced perfusion in the distal regions of the flap.

Overall, results imply that the vertical orientation and removal at 60 hours may preserve vascularity better than the horizontal orientation with Day-7 removal, although flap survival was not significantly different between groups. Prolonged hemostatic net application could potentially increase ischemia in low-vascularity areas but may also enhance immobilization, supporting blood supply through a graft-like inosculation mechanism. Thus, the lack of significant differences between early and late suture removal days is understandable.

Analysis of the distal SPY scores revealed that the lowest values were at 60 hours, and the highest at 7 days in all groups, with a significant increase between these points. Because the distal area is prone to necrosis, the hemostatic net did not produce a significant change in circulation here. Given that neovascularization is gradual, the hemostatic net's limited effect on early circulation suggests it may not be useful for immediate flap salvage.

The primary causes of skin graft failure are hematoma, infection, seroma, and shear forces.^15^ Fixation methods such as tie-over sutures, negative pressure, and tissue adhesives are essential to maintain immobilization and prevent graft loss.^16^ The hemostatic net enhances fixation, reduces dead space, and prevents fluid accumulation, potentially promoting revascularization from the base through inosculation and neovascularization.

In our study, a 6-cm section from the flap base along the midline was analyzed microscopically for new vessel formation, revealing a significant decrease in vessel counts from proximal to middle to distal in all groups. Group 5 (control) had lower vessel counts across all areas compared with the hemostatic net groups, indicating that the fixation and immobilization provided by the net promoted vessel formation. However, this increase was insufficient to prevent distal necrosis, which likely explains why the hemostatic net did not significantly impact flap survival.

Although no statistical difference was found between 60-hour and 7-day fixation times, the rapid metabolism of rats may obscure differences that might appear in clinical studies with humans. Other studies have shown that recontacting the flap with its original vascular bed improves survival rates, with survival increasing as contact duration with the vascular bed grows.^17-19^ In this study, the rat dorsal flap was returned to its original bed, whereas in facelifts, flaps are typically advanced slightly before fixation. Advancing and fixing the flap, rather than returning it directly to its original bed, might yield different results and could be a limitation in modeling the facelift flap scenario.

The modified McFarlane dorsal skin flap incorporates the panniculus carnosus and this situation may be limited in reflecting hemostatic net applications involving subcutaneous dissection. This anatomical distinction highlights the challenges of translating results from rodent models to human applications, where variations in tissue properties and healing dynamics must be carefully considered. Rodent skin exhibits differences in vascular density, wound healing dynamics, and tissue elasticity compared to human skin, which may influence the applicability of our findings.

The tension of the hemostatic net sutures, determined by the surgeon, is another limitation, and this tension can increase due to edema in the early postoperative period. In cases where excessive tension is applied or excessive postoperative edema occurs, both the orientation and duration of the hemostatic net may become significant for skin flap survival.

The primary reason for killing the animals on Day 20 of our study was to observe the effect of the hemostatic net on collagen formation; however, no significant difference was found between the groups. Increasing the number of animals might have allowed for a better evaluation of the effect of the hemostatic net on collagen production. No significant differences were observed between the groups regarding edema, inflammation, and hemorrhage. Killing the animals on Day 20 might have been a reason for the delay in observing changes in these parameters.

Kaufman et al demonstrated that applying local pressure can partially salvage the typically necrotic distal tip of the McFarlane flap by enhancing contact with the graft bed.^20^ Similarly, the hemostatic net provides improved contact through sutures. In this study, the hemostatic net groups showed increased new vessel formation compared with the control, supporting this mechanism; however, there was no significant evidence that the hemostatic net improves survival in the distal part of the flap.

Unlike previous studies that primarily focus on clinical observations, this research provides quantitative data on perfusion and neovascularization obtained by an advanced imaging technique, namely SPY fluorescence angiography, and histological analyses.

## CONCLUSIONS

The findings of this study suggest that although the hemostatic net may enhance vascularization at the flap base, it does not significantly impact overall flap survival or prevent distal necrosis in the modified McFarlane dorsal skin flap model. Although the hemostatic net facilitates better contact and fixation, which may be beneficial in clinical settings, its efficacy in improving flap survival under these experimental conditions remains limited. Further studies are necessary to explore potential clinical benefits and alternative approaches for enhancing distal flap vascularization and survival.
